# Phenology of five tick species in the central Great Plains

**DOI:** 10.1371/journal.pone.0302689

**Published:** 2024-05-09

**Authors:** Eric Ng’eno, Abdelghafar Alkishe, Daniel Romero-Alvarez, Kellee Sundstrom, Marlon E. Cobos, Hallee Belgum, Abigail Chitwood, Amber Grant, Alex Keck, Josiah Kloxin, Brayden Letterman, Megan Lineberry, Kristin McClung, Sydney Nippoldt, Sophia Sharum, Stefan Struble, Breanne Thomas, Anuradha Ghosh, Robert Brennan, Susan Little, A. Townsend Peterson

**Affiliations:** 1 Biodiversity Institute, University of Kansas, Lawrence, Kansas, United States of America; 2 Faculty of Health Sciences, Emerging and Neglected Diseases, Ecoepidemiology and Biodiversity Research Group, Universidad Internacional SEK (UISEK), Quito, Ecuador; 3 College of Veterinary Medicine, Oklahoma State University, Stillwater, Oklahoma, United States of America; 4 Department of Biology, Pittsburg State University, Pittsburg, Kansas, United States of America; 5 Department of Biology, University of Central Oklahoma, Edmond, Oklahoma, United States of America; University of Oklahama Norman Campus: The University of Oklahoma, UNITED STATES

## Abstract

The states of Kansas and Oklahoma, in the central Great Plains, lie at the western periphery of the geographic distributions of several tick species. As the focus of most research on ticks and tick-borne diseases has been on Lyme disease which commonly occurs in areas to the north and east, the ticks of this region have seen little research attention. Here, we report on the phenology and activity patterns shown by tick species observed at 10 sites across the two states and explore factors associated with abundance of all and life specific individuals of the dominant species. Ticks were collected in 2020–2022 using dragging, flagging and carbon-dioxide trapping techniques, designed to detect questing ticks. The dominant species was *A*. *americanum* (24098, 97%) followed by *Dermacentor variabilis* (370, 2%), *D*. *albipictus* (271, 1%), *Ixodes scapularis* (91, <1%) *and A*. *maculatum* (38, <1%). *Amblyomma americanum*, *A*. *maculatum and D*. *variabilis* were active in Spring and Summer, while *D*. *albipictus and I*. *scapularis* were active in Fall and Winter. Factors associated with numbers of individuals of *A*. *americanum* included day of year, habitat, and latitude. Similar associations were observed when abundance was examined by life-stage. Overall, the picture is one of broadly distributed tick species that shows seasonal limitations in the timing of their questing activity.

## Introduction

The Central Great Plains region covers a large and biologically diverse area that is home to a variety of tick species as well as abundant wildlife [1]. Ticks are carriers of a variety of pathogens that can cause serious diseases in humans and other animals [2]. Numbers of human cases from tick-borne diseases such as tularemia, Rocky Mountain spotted fever (RMSF), ehrlichiosis, anaplasmosis, and Lyme borreliosis have increased in recent years [3,4]. These increases in case rates have been attributed to changes in climate conditions [5], tick range expansions [2], habitat and land use changes [6], and host population dynamics [7]. With the ongoing processes of climate change [8], and land-use change [9], in the Great Plains region, it is important to understand the life cycles, population dynamics, and interactions between the environment and different tick species of public health importance.

The phenology of ticks in the Central Great Plains is likely influenced by an interplay of factors, including temperature, humidity, vegetation type, and host availability [10], given the region’s distinctive blend of grasslands, forests, and agricultural landscapes. Different tick species’ seasonal prevalence, abundance, and distribution, on these landscapes might offer important clues to their ecological preferences, and likely responses to change in climate conditions [11]. Since ticks are dependent on vertebrate hosts for survival and reproduction, their phenology is also closely linked to the actions and life cycles of their hosts [12]. Ticks adapt to these differences by displaying patterns of activity, questing behavior, and lifecycle transitions throughout the course of the year [13].

A few previous studies have tracked the phenology of ticks in the central Great Plains (e.g., in Kansas, Oklahoma, and Missouri) over the past 10 years [10,14–17]. Recent studies in southeastern Kansas, and the Flint Hills region of eastern Kansas and north-central Oklahoma, evaluated seasonality and diversity of tick species in the area [4,10,18]. The most common and broadly distributed tick species were *A*. *americanum*, *A*. *maculatum*, *Dermacentor variabilis*, and *Ixodes scapularis*, a diversity of tick species that raises significant concerns about the potential for transmission of tick-borne diseases [4,19]. Here we summarized phenological aspects of tick communities documented in longitudinal multisite study of questing ticks (2020–2022), from across the states of Kansas and Oklahoma, and identified factors associated with questing activities of *A*. *americanum* in the region.

## Methods

### Study sites

**Fig 1:**
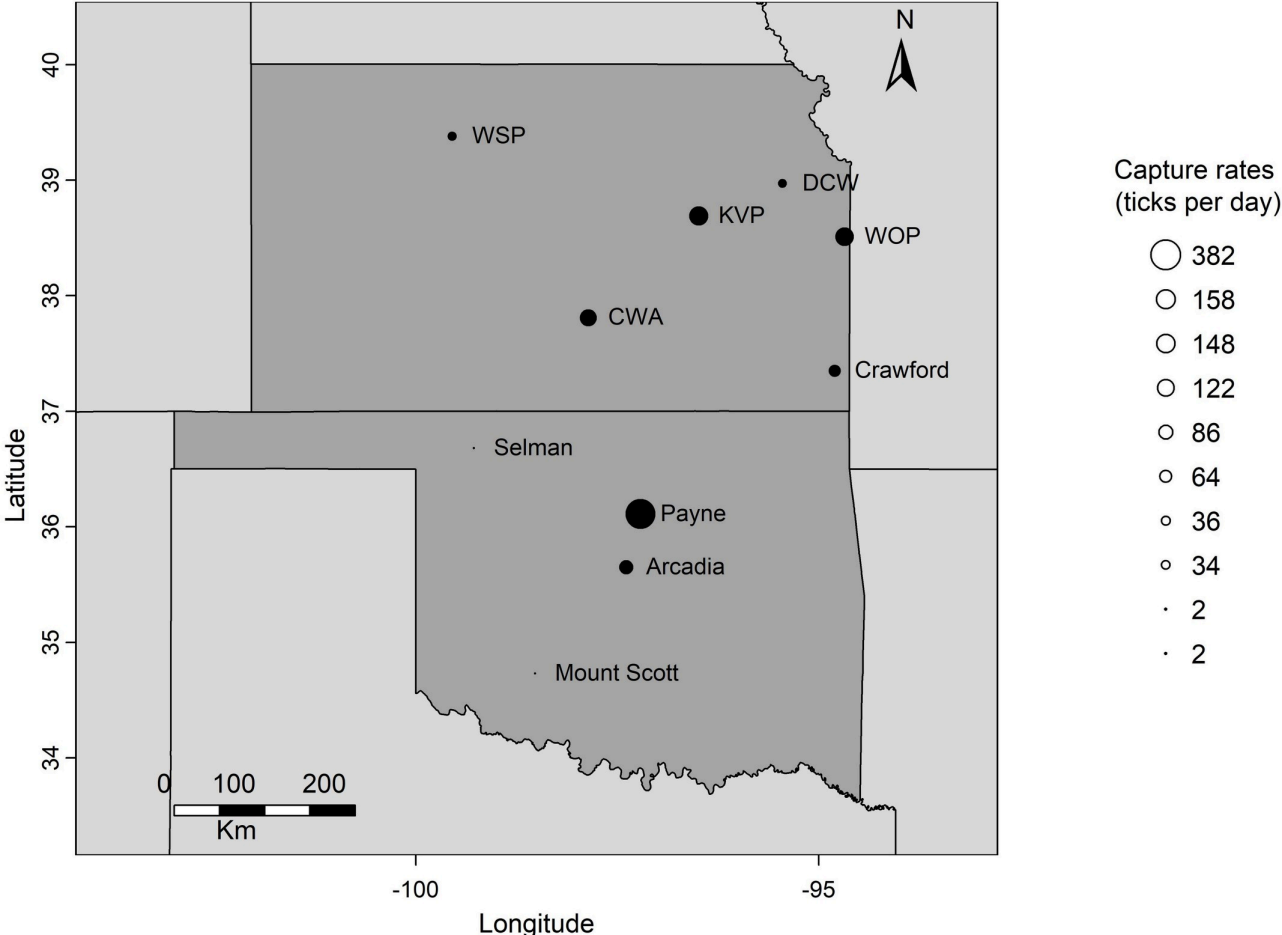
Map of Kansas and Oklahoma, showing the locations of the 10 sites where ticks were sampled as part of this study. Some of the site names have been abbreviated or nicknamed: Lake Arcadia (Arcadia), Cheney Wildlife Area (CWA), Deer Creek Wildlife Area (DCW), Kanza View Park (KVP), Rutlader Wildlife Area (WOP) and Webster State Park (WSP) Circle size indicates the number of ticks captured, normalized to the number of days of sampling, which ranged from 2 ticks/day to 382 ticks/day across the sites.

We collected ticks at 10 publicly accessible sites distributed across Kansas and Oklahoma so that sampling can be repeated easily in the future (Fig 1). The sites represent different ecosystems and habitats, and were chosen to cover as best as possible the diversity of ecosystems present across the region. Sites are described in detail in Table 1.

**Table 1:** Description of sampling sites in Kansas and Oklahoma, August 2020 to September 2022.

State	County	Sites	Ecoregion*	Coordinates	Habitat	Description
Kansas	Reno	Cheney wildlife area (rural)	Wellington-Mcpherson Lowland	37.81, -97.86	Pasture	A mix of non-prairie grasses and small shrubs.
					Wooded	A mix of shrubs and wooded plants, including honeysuckle, cedar, oak, and walnut.
	Crawford	Crawford (rural near an artificial wetland)	Cherokee Plains	37.35, -94.80	Pasture	Mixed prairie grasslands, including, big bluestem and switchgrass,
					Wooded	A mix of shrubs and wooded plants including bush honeysuckle, rough-leafed dogwood, dwarf sumac, salt cedar, pin and red oak.
	Douglas	Deer Creek wildlife area (rural)	Osage Cuestas	38.97, -95.45	Pasture	A mix of non-prairie grasses and small shrubs.
					Wooded	A mix of shrubs and wooded plants, including honeysuckle, cedar, oak, cottonwood, elm, and walnut.
	Morris	Kanza view park (rural)	Flint Hills	38.69, -96.49	Pasture	A mix of non-prairie grasses and small shrubs.
					Wooded	A mix of shrubs and wooded plants including honeysuckle, cedar, cottonwood and walnut.
	Miami	Rutlader wildlife area (rural)	Wooded Osage Plains	38.51, -94.68	Pasture	A mix of distinct species of grass. Non prairie grass
					Wooded	A mix of shrubs and wooded plants, mainly cedar and juniper.
	Rooks	Webstar state park (rural)	Rolling Plains and Breaks	39.38, -99.55	Pasture	Secondary prairie grass.
					Wooded	A mix of wooded plants, mainly pine, cedar, and juniper.
Oklahoma	Payne	Payne (rural)	Cross Timbers Transition	36.11, -97.21	Pasture	A mix of grasses and small shrubs. Non prairie grass.
					Wooded	A mix of shrubs and wooded plants, including honeysuckle. Dominated by mixed oak and cedar.
	Oklahoma	Lake Arcadia (peri urban)	Prairie Tableland	35.65, -97.39	Pasture	Mixed-grass plains including little bluestem, big bluestem, and Indian grass
					Wooded	Predominantly a mixture of post oak, blackjack oak, hackberry, and eastern red cedar trees
	Woods	Selman (rural)	Tablelands	36.68, -99.28	Pasture	Mixed-grass plains, common grasses include little bluestem, sand bluestem, silver bluestem, Indian grass, switch grass, grama grasses, and buffalo grass
					Wooded	Mixture of hackberries, elms, cottonwood, chittamwood, and willow
	Comanche	Mount Scott (rural, part of the Wichita Mountains Wildlife Refuge)	Broken Red Plains	34.73, -98.52	Pasture	Mixed prairie grasslands, common grasses include buffalo, grama short-grasses, bluestems, Indian grass, and switch-grass tallgrasses
					Wooded	Scrubby forest of mixed oaks

* Level IV Ecoregions of the Continental United States. Available at https://gaftp.epa.gov/EPADataCommons/ORD/Ecoregions. Accessed on 9th February 2024.

### Sampling frequency

**Fig 2:**
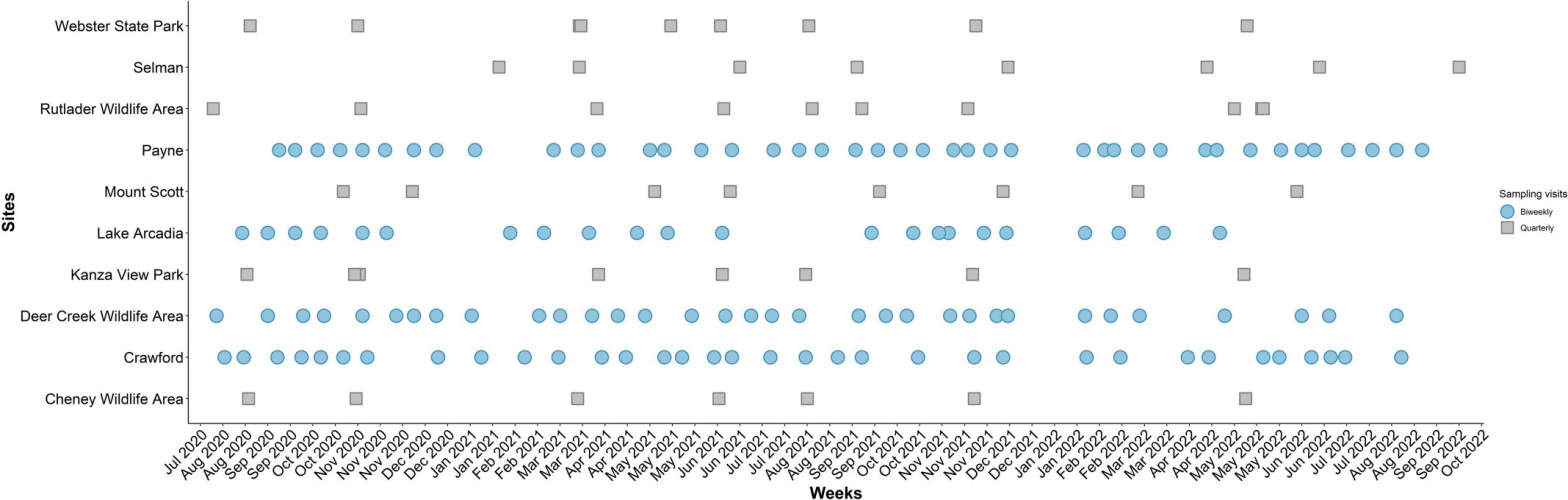
Sampling frequency across sites over time (August 2020 to September 2022). Blue circles represent sites that were sampled approximately every after two weeks, whereas gray squares represent sites that were sampled every quarter.

Between 1 August 2020 and 19 September 2022, we conducted tick sampling approximately on a biweekly basis in Crawford and Douglas counties in Kansas, and in Oklahoma and Payne counties in Oklahoma; we sampled the remaining sites on a quarterly basis (Fig 2). Biweekly sites were selected on feasibility for frequent and year-round sample collection. The data from the Payne site for *A*. *americanum* have been reported previously [18]. In this analysis, however, we report on other tick species which were identified at that site, evaluate phenological patterns across the broader two-state region, and use *A*. *americanum* to draw comparisons with the other sites.

### Field collections

To standardize collection of ticks across sites, sampling at each site was conducted by trained study staff. Collectors used dragging and flagging, and carbon-dioxide trapping techniques to avoid biases that are associated with reliance on one or a few methods. At each site, we identified two 50 m x 50 m sampling blocks. Each block included one half of open (pasture) habitat and another half of wooded habitat. One sampling block was used for dragging and flagging, and the other for dry-ice trapping. The transects were marked and were visited throughout the study period. We conducted field collections on non-rainy days with wind speed <15 miles per hour and temperature between 2–32°C during late mornings and afternoons when dew was not present. Temperature, humidity, and wind measurements were collected at the start and end of sampling. Winter collections were performed in the afternoon, when the temperature was above 2°C and there was no snow.

#### Tick collection by dragging

Tick drags consisted of a 1 m^2^ piece of flannel material weighted by a 2.54 cm (round) x 91.44 cm (long) wooden dowel on either side, affixed to a long rope. We used drags in open fields and grassy meadows that did not present obstacles or risk of frequent snags. To drag for ticks, we placed the flannel fabric lightly on the vegetation at the edge of one corner of the transect and walked slowly forward, allowing the drag to trail behind, making sure that the drag contacted as much of the ground as possible. In open half of each 50 m x 50 m transects, drags were performed along straight lines 1 m apart while in wooded half, drags were performed in wavy lines approximately 1 m apart as allowed by the density of trees. In total we performed a total of 25 lines of dragging in each half of the sampling block. At the end of every dragging line, we inspected the drag, collected any ticks that had accumulated and stored them in tubes. We covered the entire block by walking slowly, over the course of ~60–80 minutes.

#### Tick collection by flagging

Tick flags consisted of 0.8 m x 0.8 m x 1.13 m triangles of flannel material attached along one edge to a wooden dowel (1.28 cm round x 121.92 cm long). To ensure that the flag contacted as much of the brushy vegetation, we waved the flag over the vegetations from the ground to 1 m above the ground. About every 2 minutes, we inspected the drag, collect any ticks that had accumulate and stored them in tubes. We covered the block transect by walking slowly, over the course of ~60–90 minutes.

#### Tick collection by dry ice trapping

For dry ice trapping we used “tick traps” consisting of 0.3 m x 0.3 m cardboard squares rimmed with masking tape (sticky side up), and with dry ice placed in the center of the cardboard square. In total, we placed sixteen traps across the open and wooded half of each 50 m x 50 m transects. The traps were placed in a grid pattern ~10 m apart. We left the traps in place for at least 2 hours before retrieval and collection of any ticks present on the traps.

### Field sample processing and data documentation

Using forceps, we transferred collected ticks into specimen tubes with 70% ethanol. We labeled the tubes and stored them at -80°C. We recorded field data including (1) collection date, site name, GPS coordinates, field arrival and departure times, ambient temperature, relative humidity, wind speed, ambient light, tick species, and transect or trap number; all transects, and trap locations were documented via GPS-enabled digital photographs.

### Laboratory identification

Using a variety of optical equipment, we identified life stage, sex, and species based on morphology using standard dichotomous keys (Strickland 1976, Keirans and Durden 1998).

### Data processing and statistical testing

We assembled a raw dataset linking the number of collected ticks, field data, species, sex and life stages of the ticks. We rounded geographic coordinates to 2 decimal places, such that each site was referred to a single set of geographic coordinates. We then combined male and female as adult ticks and excluded from the dataset records from the pilot phase of the project.

### Data analysis

We summarized sampling frequency, number of collected ticks, life stages and species identified across the sampling period. We created counts and frequencies and visualized them as maps, tables, and charts summarizing sampling frequencies, number of ticks of different life stages and species collected over time at each site. We identified peak activity times as the months with the highest number of ticks after standardizing for sampling effort. We standardized for sampling by dividing the number of ticks collected at a site by the proportion of actual and expected sampling events for that site.

We analyzed factors associated with abundance of *A*. *americanum*, in view of its high numbers of overall and life-stage specific ticks collected. Factors including day of the year, latitude, longitude, and habitat (wooded area versus open area), were explored for overall *A*. *americanum* abundance and abundances of each life-stage with multivariate statistical models. We fitted multiple Poisson generalized linear mixed-effect models that assembled different combinations of predictor variables and chose “best” models as those with the lowest Akaike information criterion (AIC) value, that is, the model that explained most of the variance in the training data with the fewest number of predictor variables [20]. We used site visit frequency nested within year of sampling as a random intercept to account for potential variation owing to differences in frequency of sampling events across the sites and years. We standardized day of the year, latitude, and longitude predictor variable, by subtracting respective means and dividing by the standard deviation. The models were generated using the lme4 package version 1.1–34 in R, version 4.2.2 [21,22].

### Ethics

Tick samples were collected from public sites, with administrative approvals from managers of the parks and wildlife areas as necessary.

## Results

During the period 1 August 2020 to 19 September 2022, a total of 181 sampling visits at 10 sites across Kansas and Oklahoma states were conducted: 41 visits (22.7% of total) in 2020, 93 (51.4%) in 2021, and 47 (25.9%) in 2022. Payne had the highest sampling frequency (42 visits), followed by Crawford (34 visits) (Fig 1).

In total, 25168 individual ticks were collected. Of these, 16046 (63.8%) were from Payne, 2168 (8.6%) from Crawford, and 1898 (7.5%) from Lake Arcadia (Table 2). Among the ticks collected, *A*. *americanum* was the most common species identified (24098; 95.7%), followed by *D*. *variabilis* (370; 1.5%), *D*. *albipictus* (271;1.1%), *I*. *scapularis* (91; 0.4%), and *A*. *maculatum* (38; 0.2%). Three hundred (1.2%) larvae were not characterized to the species level.

**Table 2:** Distribution of number of collected ticks by species, life stage and collection sites.

		Payne	Crawford	Lake Arcadia	Rutlader Wildlife Area	Kanza View Park	Deer Creek Wildlife Area	Cheney Wildlife Area	Webster State Park	Mount Scott	Selman	Overall
		(N = 16046)	(N = 2168)	(N = 1898)	(N = 1478)	(N = 1260)	(N = 1113)	(N = 851)	(N = 323)	(N = 17)	(N = 14)	(N = 25168)
Species	Life stage											
***A*. *americanum***	**Overall**	**15984 (99.6%)**	**1683 (77.6%)**	**1891 (99.6%)**	**1431 (96.8%)**	**1238 (98.3%)**	**857 (77.0%)**	**805 (94.6%)**	**186 (57.6%)**	**16 (94.1%)**	**7 (50.0%)**	**24098 (95.7%)**
	Larvae	8564	0	0	21	173	136	54	60	0	0	9008
	Nymph	5534	881	606	840	755	306	324	34	9	2	9291
	Adult	1886	802	1285	570	310	415	427	92	7	5	5799
***D*. *variabilis***	**Overall**	**0 (0.0%)**	**181 (8.3%)**	**2 (0.1%)**	**16 (1.1%)**	**14 (1.1%)**	**102 (9.2%)**	**11 (1.3%)**	**38 (11.8%)**	**0 (0.0%)**	**6 (42.9%)**	**370 (1.5%)**
	Larvae	0	0	0	0	0	39	0	0	0	0	39
	Nymph	0	41	0	0	0	0	1	0	0	0	42
	Adult	0	140	2	16	14	63	10	38	0	6	289
***D*. *albipictus***	**Overall**	**0 (0.0%)**	**0 (0.0%)**	**0 (0.0%)**	**27 (1.8%)**	**3 (0.2%)**	**118 (10.6%)**	**24 (2.8%)**	**99 (30.7%)**	**0 (0.0%)**	**0 (0.0%)**	**271 (1.1%)**
	Larvae	0	0	0	27	3	118	24	99	0	0	271
	Nymph	0	0	0	0	0	0	0	0	0	0	0
	Adult	0	0	0	0	0	0	0	0	0	0	0
***I*. *scapularis***	**Overall**	**62 (0.4%)**	**0 (0.0%)**	**5 (0.3%)**	**2 (0.1%)**	**1 (0.1%)**	**20 (1.8%)**	**1 (0.1%)**	**0 (0.0%)**	**0 (0.0%)**	**0 (0.0%)**	**91 (0.4%)**
	Larvae	0	0	0	0	0	0	0	0	0	0	0
	Nymph	0	0	0	0	0	0	0	0	0	0	0
	Adult	62	0	5	2	1	20	1	0	0	0	91
***A*. *maculatum***	**Overall**	**0 (0.0%)**	**4 (0.2%)**	**0 (0.0%)**	**2 (0.1%)**	**4 (0.3%)**	**16 (1.4%)**	**10 (1.2%)**	**0 (0.0%)**	**1 (5.9%)**	**1 (7.1%)**	**38 (0.2%)**
	Larvae	0	0	0	0	0	0	0	0	0	0	0
	Nymph	0	0	0	0	0	0	0	0	0	0	0
	Adult	0	4	0	2	4	16	10	0	1	1	38
**Not defined**	**Overall**	**0 (0.0%)**	**300 (13.8%)**	**0 (0.0%)**	**0 (0.0%)**	**0 (0.0%)**	**0 (0.0%)**	**0 (0.0%)**	**0 (0.0%)**	**0 (0.0%)**	**0 (0.0%)**	**300 (1.2%)**
	Larvae	0	300	0	0	0	0	0	0	0	0	300
	Nymph	0	0	0	0	0	0	0	0	0	0	0
	Adult	0	0	0	0	0	0	0	0	0	0	0

Separating ticks collected by life stage, 9618 (38.2%) were larvae (although this life stage may have been under-counted owing to their small size), 9333 (37.1%) were nymphs, and 6217 (24.7%) were adults (Table 2). Nymphs were the most common life stage in *A*. *americanum* (9291, 37.4%), followed by larvae (9008, 36.2%). For *D*. *variabilis*, we collected 289 (78.1%) adults and 42 (11.4%) nymphs. Only adult ticks were collected for *A*. *maculatum* and *I*. *scapularis*; only larvae were collected for *D*. *albipictus*.

*Amblyomma americanum* was the most common species identified at all sites (Table 2). *Dermacentor variabilis*, the second commonly observed species, was collected from all sites except Mount Scott and Payne. *Dermacentor albipictus* was observed at Cheney Wildlife Area, Deer Creek Wildlife Area, Kanza View Park, Rutlader Wildlife Area, and Webster State Park. *Ixodes scapularis* was observed at Cheney Wildlife Area, Deer Creek Wildlife Area, Lake Arcadia, and Payne, whereas *A*. *maculatum*, the least common species, was collected from all sites except Lake Arcadia, Payne, and Webster State Park.

**Fig 3:**
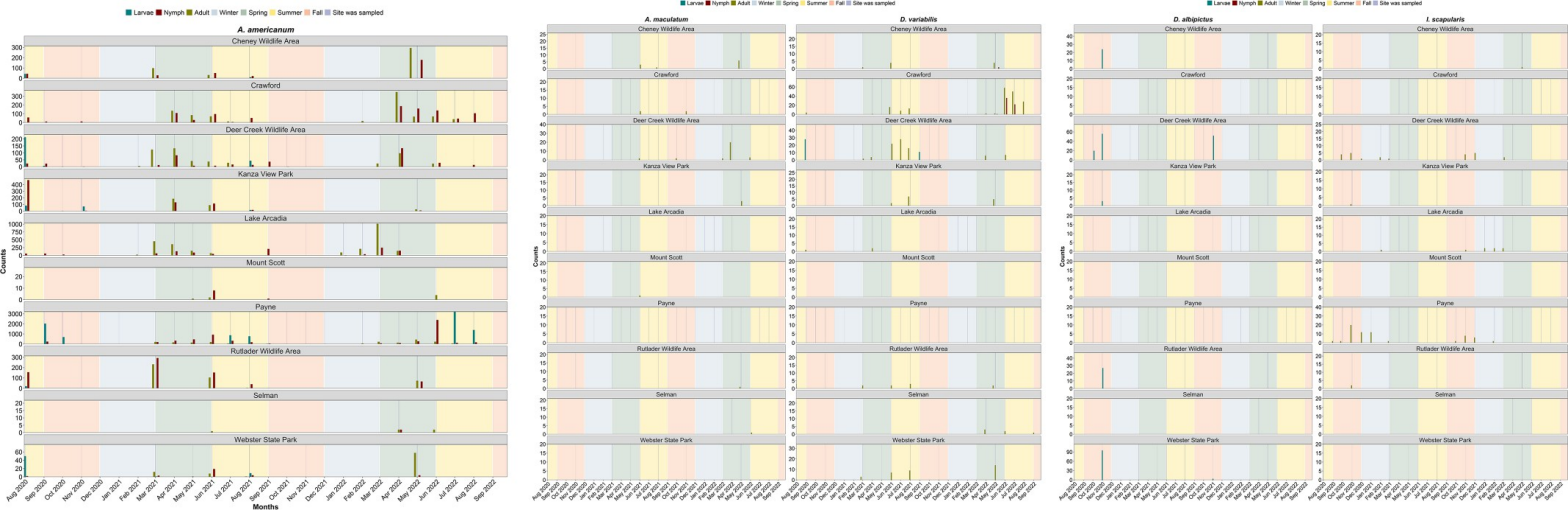
**a-c**. Phenology of identified tick species (*Amblyomma americanum*, *A*. *maculatum*, *Dermacentor variabilis*, *D albipictus*, and *Ixodes scapularis*) and their life stages by sampling sites during the period August 2020 to September 2022. The blue lines represent sampling dates while seasons are represented with background colors of the plot.

Across all sites, *A*. *americanum*, *A*. *maculatum* and *D*. *variabilis* occurred during the Spring and Summer (Fig 3A and 3B). In sharp contrast, *D*. *albipictus* and *I*. *scapularis* were observed only in the Fall, and Fall and Winter, seasons, respectively (Fig 3C). There were sporadic detections of a few nymphal and adult individuals of *A*. *americanum* in January and February, in Crawford, Deer Creek Wildlife Area, Lake Arcadia, and Payne (Fig 3A).

Among sites that were visited every after two weeks, peak questing activity times for *A*. *americanum* nymphs and adults occurred early March in Lake Arcadia, late March to the beginning of April in Crawford and Deer Creek Wildlife Area, and late May to early June in Payne (Fig 3A). Larvae, which were collected only at Payne, peaked in late June to early July. *Dermacentor variabilis* peak questing activity of adults and nymphs was in June through July at Crawford and Deer Creek Wildlife Area, where most individuals were collected (Fig 3B). During the study period, the peak of *D*. *variabilis* activities was in July of 2021 at Deer Creek Wildlife Area, whereas at Crawford the peak was in June of 2022. *Ixodes scapularis* adult questing activity peaked in late October at Deer Creek Wildlife Area and Payne (Fig 3C). A higher peak was observed in Payne in October 2020. There were too few observations of *A*. *maculatum* and *D*. *albipictus* to determine questing seasonality in much detail (Fig 3B and 3C).

**Fig 4:**
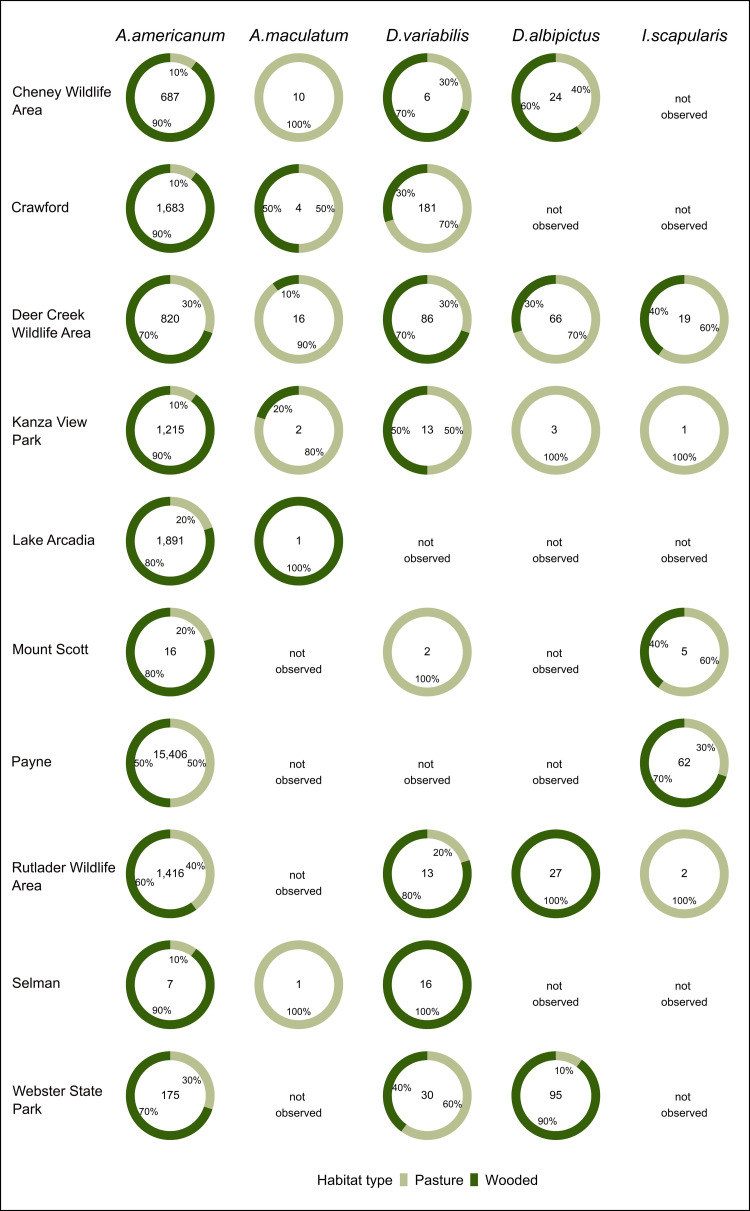
Distribution of identified tick species (*Amblyomma americanum*, *A*. *maculatum*, *Dermacentor albipictus*, *D variabilis*, and *Ixodes scapularis*) in wooded and pasture habitats by sampling sites during the period August 2020 to September 2022.

Assessing frequency of collection of different tick species by habitat, more individuals of *A*. *americanum* were collected in wooded habitats across all sites (Fig 4). Fewer individuals per site made it challenging to draw conclusions regarding preferred habitats for the other tick species observed.

**Fig 5:**
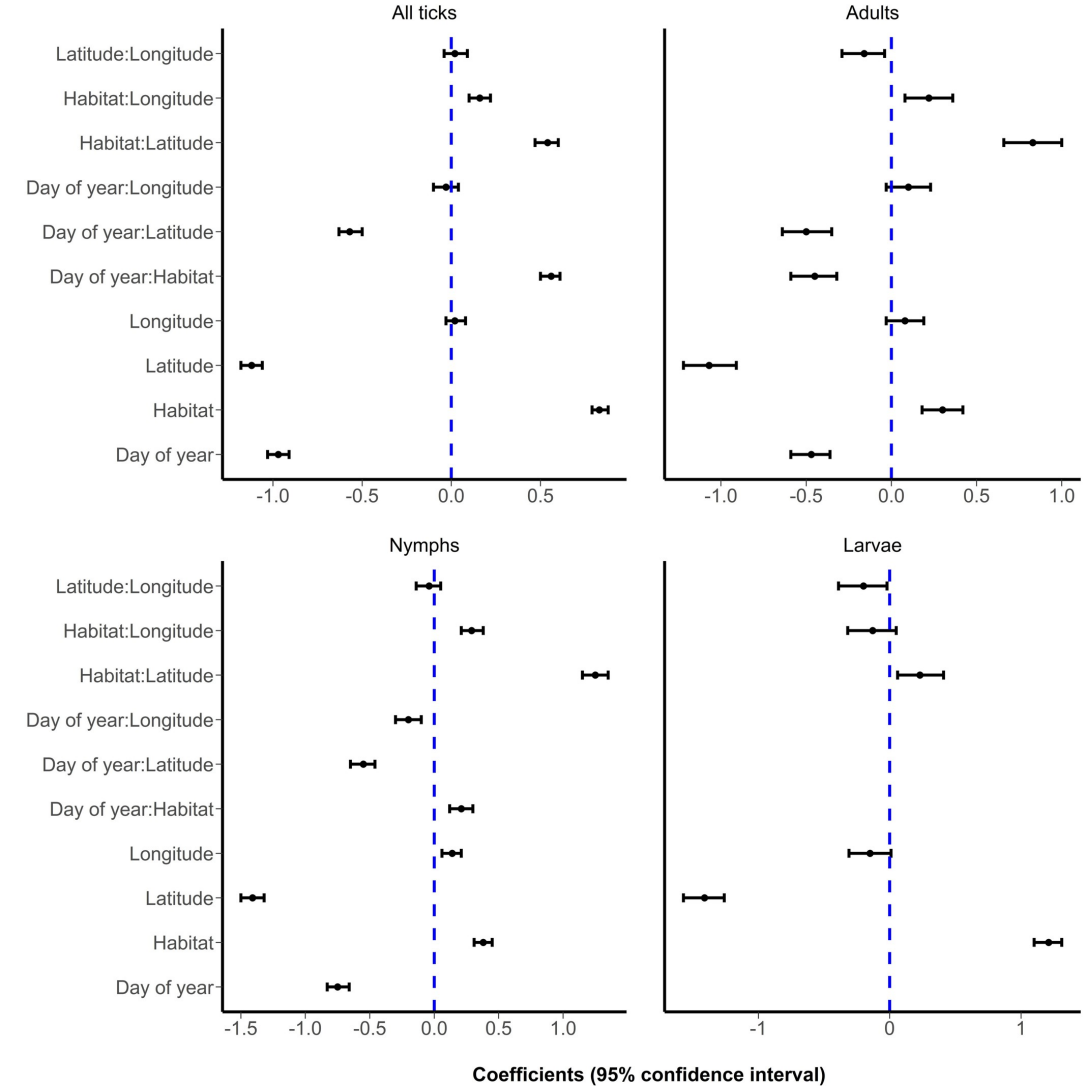
Estimated coefficients and 95% confidence intervals of models assessing association between environmental factors and abundances of *Amblyomma americanum* for all individuals combined, and for each life-stage separately. Models were generated using data for all sites during the period August 2020 to September 2022. Habitat represents wooded habitat.

Our statistical analyses of factors associated with observed numbers of questing *A*. *americanum* showed significant overall (all ticks) effects of day of year, latitude, and habitat (Fig 5). The number of individuals of *A*. *americanum* increased (estimate 0.83, 95% CI 0.79–0.88) in wooded areas and decreased with increase in the day of year [-0.97, CI -1.03 –-0.91)] and latitude (-1.12, CI -1.18 –-1.06). The nature of these associations was similar when assessed by life-stage. Numbers of nymphs were also associated with longitude (0.14, CI 0.06–0.21), increasing with increase in longitude. Day of year and habitat interactions were associated with increased abundance for all ticks and nymphs and decreased abundance for adults. Interactions between day of year and latitude were associated with decreased numbers of all ticks, and life-stage-specific abundances as well. Interaction of day of year and longitude was associated with decreased number of nymphs. Habitat interactions with latitude, and longitude were associated with increased abundance of all and life-stage specific ticks.

## Discussion

This analysis was designed to fill gaps in knowledge of tick communities in the central Great Plains. Ticks were collected from multiple sites in the states of Kansas and Oklahoma. Each site included both wooded areas and pasture habitat types. In accord with recent recommendations [23], we sampled the ticks using multiple sampling methods (flags and drags, tick traps) to maximize our probability of detecting all questing tick species present in the sites.

We identified five tick species of public health importance. Previous studies conducted at sites near, and including some of our sites, have identified much the same set of species [4,10,17–19]. *Amblyomma americanum* was the most abundant, comprising approximately 97% of all individual ticks collected. The five tick species varied by site of collection and showed distinct phenologies, with *A*. *americanum*, *A*. *maculatum*, and *D*. *variabilis* occurring in the Spring and Summer, and *D*. *albipictus* and *I*. *scapularis* observed in late Fall and Winter. Peak activity timings varied by site and species. At Lake Arcadia, for example, questing activities of nymphs of *A*. *americanum* peaked in March which deviated from findings of previous studies in the area which reported peaks in the months of April and May [24,25], and May and June [17,26]. However, peak questing timing at the other sites were consistent with those of recent studies; late March-early April at Crawford and Deer Creek wildlife area [4] and late May-early June in Payne [26]. Peaks for *D*. *variabilis* and *I*. *scapularis* did not deviate from the findings of previous studies the area [4,17,24]. Factors associated with the numbers of individuals of *A*. *americanum* were day of year, latitude, and wooded habitat.

Previous studies on phenology and activity of ticks in the region could help to contextualize our results. Comparisons of *A*. *americanum* activities with the historical questing patterns [25,26], shows a general earlier shift in peak timings of up to 2 months across the sites. The changes in peak timings could be attributed in part to warming climate [10], which has been associated with north-and-westward ranges expansion of the tick [27–30]. Phenology studies in warmer southeastern parts of United States have reported peak activity between March and April for *A*. *americanum* [31] which suggests that as warmer conditions become established in these areas early peak questing timings could become common. Milder winter in the region owing to the warming climate, could also see increasing questing activities of *A*. *americanum* during the months of January and February [32]. While we observed unimodal nymphal peak questing activity in this and another recent study in the area [4], past studies and recent study in the southeastern have reported bimodal patterns in nymphal questing activities [25,26,31,33]. The occurrence of a second questing peak has been attributed to early larvae to nymph molting with newly molted nymphs actively seeking for host before winter onset [34]. Early winter onset in the region—when compared to the past period—[10] could have driven early nymphal diapause, however this could change with the warming conditions.

Our assessment of factors associated with the abundance of members of *A*. *americanum* showed a positive relationship between the number of ticks and wooded areas. Wooded habitats with leaf litter cover and understory can provide cooler and humid conditions that can enhance survival especially of *A*. *americanum* nymphs [35,36]. Woody habitat has also been associated with increased vertebrate host populations, such as white-tailed deer (*Odocoileus virginianus*), considered a preferred host of *A*. *americanum* [37].

Latitude, on the other hand, was inversely related with the abundance of *A*. *americanum*. The general decrease in tick abundance southward can be attributed to increasing temperatures associated with climate change in the southeastern United States [38]. Prolonged hot and dryer conditions have been shown to limit survival of *A*. *americanum* in the open habitats [39]. However, our exploration of the interaction between latitude and wooded habitats showed that forested areas further south had higher ticks’ abundance suggesting woody habitats in southeast may be providing microhabitats conducive for higher populations of *A*. *americanum*. Woody plant encroachment has been documented across the central Great Plains region [40], as such tick populations could increase in tandem with the extent of forested areas in the region [41]. We also observed a negative association between abundance of *A*. *americanum* and day of year, which we attribute to decreasing photoperiod in latter days of the year.

A tick species of particular interest and importance that was detected in our sampling is *I*. *scapularis*, which is considered the primary vector of the Lyme disease pathogen (*Borrelia burgdorferi)* in the northeastern, mid-Atlantic, and upper north-central regions of the United States, as well as along parts of the Pacific Coast [42,43]. Although recent modeling efforts have suggested that populations of this species are either absent or rare in the central Great Plains [44], the model-based results proved to be artifactual [45,46]. Populations of the species have indeed been documented in the region, sparse but broadly present [4,47], and apparently increasing in population [16]. This study and other recent studies [4,32] provide a clearer explanation for these contrasting perceptions regarding this tick species along the western periphery of its distributional area: it is present at low densities, but it is also questing actively only in the Fall and Winter months, and not in the Spring and Summer like most of the ticks in the region. Clearly, phenological aspects of this tick species are complex, and are in need of much additional documentation [31,48–50].

These findings are reported with the following limitations. While the study was both intensive (e.g., biweekly sampling) and extensive (e.g., sites scattered across two large states) with sampling based on three modes of accumulating ticks, we did not find life stages of three of the five tick species, likely owing to differences in their questing behavior [51,52]. Surveys including detailed sampling of mammals and other vertebrate hosts could potentially improve detection of these life stages. Broader-scale sampling could also detect other, more specialized tick species at our study sites. The prevalence and abundance of larval ticks was underestimated likely due to their small size and the difficulty of detecting and collecting them. Weather conditions limited sampling during winter months at certain sites which could have impacted detection of species active during that time.

Overall, with the benefit of multiple sites across the states of Kansas and Oklahoma, we highlight five species of ticks in the area with *A*. *americanum* as the most common. We show potentially evolving phenology and activity of *A*. *americanum* potentially owing to the changing climate in the region. Continued monitoring of the evolving distributions and abundance patterns is important to inform control strategies of these human and veterinary important ticks in the area.

## Supporting information

S1 File (ZIP)

S1 Data (CSV)
